# 8-armed octopus: Evaluation of clinicopathologic prognostic factors of urothelial carcinoma of the upper urinary system

**DOI:** 10.3906/sag-1805-51

**Published:** 2019-02-11

**Authors:** Sümeyye EKMEKCİ, Ülkü KÜÇÜK, Yelda DERE, Ebru ÇAKIR, Hatice Ceren SAYAR, Batuhan ERGANİ, Özgür ÇAKMAK, Ozan BOZKURT, Kutsal YÖRÜKOĞLU

**Affiliations:** 1 Department of Pathology, Health Sciences University Tepecik Training and Research Hospital, İzmir Turkey; 2 Department of Pathology, Faculty of Medicine, Muğla Sıtkı Koçman University, Muğla Turkey; 3 Department of Pathology, Atatürk Training and Research Hospital, Faculty of Medicine, Katip Çelebi University, İzmir Turkey; 4 Department of Urology, Health Sciences University Tepecik Training and Research Hospital, İzmir Turkey; 5 Department of Urology, Faculty of Medicine, Dokuz Eylül University, İzmir Turkey; 6 Department of Pathology, Faculty of Medicine, Dokuz Eylül University, Izmir Turkey

**Keywords:** Prognostic factors, upper tract urothelial carcinomas, upper urinary tract, urothelial carcinomas

## Abstract

**Background/aim:**

This study was designed to determine the characteristic features of upper urinary system urothelial carcinomas (UUSUCs) and to evaluate the clinicopathological parameters associated with prognosis.

**Materials and methods:**

A total of 74 cases of UUSUC were included, from three different centers. Demographic data and histopathological features such as tumor localization, concomitant tumor in the urinary system, distant metastasis with overall survival and disease-free survival obtained from the hospital records were evaluated retrospectively. Histopathologic prognostic features such as grade, perineural invasion, lymphovascular invasion, tumor necrosis, and surgical margin status were also evaluated.

**Results:**

Seventy cases (94.6%) underwent open nephroureterectomy whereas 4 cases (5.4%) had laparoscopic nefroureterectomy. Thirty-eight (51.4%) cases were located in the pelvis, 7 (9.5%) in the ureter, 29 (39.2%) both in the pelvis and ureter. Fifty-six (75.7%) cases were alive; however, 18 (24.3%) patients were found to be dead. pTa, pT1, pT2, pT3, and pT4 tumors were reported in 16 (21.6%), 13 (17.6%), 4 (5.4%), 28 (37.8%), and 13 (17.6%) patients, respectively. Histopathologically, 17 cases (23%) were low-grade, 57 cases (77%) were high-grade. Statistically significant correlation was observed between overall survival and lymph node metastasis, distant metastasis, tumor necrosis, and differentiation by univariate analysis. Only distant metastasis was statistically associated with overall survival by multivariate analysis. We found no significant relationship between disease-free survival and all parameters.**Conclusions: **Differentiation and necrosis of tumor, lymph node involvement, and presence of distant metastasis is associated with the overall survival of urothelial carcinoma of the upper urinary system.

## 1. Introduction

Urothelial carcinomas (UC) can arise in any part of the urinary tract lined by urothelium; however, the majority of cases are located in the lower tract (bladder, urethra) (1,2). Upper urinary system urothelial carcinomas (UUSUC), including renal pelvis and ureteral tumors, are known to be rare tumors, which constitute approximately 5%–10% of all UCs. The natural history and prognosis of UUSUC differ from bladder cancer (3–6). Among UUSUC, 60% of cases are invasive at diagnosis while only 15%–25% of bladder tumors are invasive at initial diagnosis. Moreover, the prognosis of UUSUC is poor and five year recurrence-free and overall survival (OS) rates are reported as 28% and 23%, respectively (1,2,7–13).

There are many studies in the literature evaluating the factors affecting the prognosis of the UC but the data about the prognostic factors of UUSUC are limited (1, 7, 9, 12-16). In this study, we evaluated the effect of clinicopathological factors including age, sex, tumor grade, tumor stage, tumor necrosis, lymphovascular invasion (LVI), perineural invasion (PNI), lymph node metastasis (LNM), and distant metastasis on OS of UUSUC.

## 2. Materials and methods

A total of 74 cases diagnosed with UUSUC from three different centers between February 2000 and December 2017 were included in the study. In patients with suspicion of UUSUC, diagnosis was obtained radiologically by using computed tomography urography which has the highest diagnostic accuracy among all of the clinically available imaging techniques. Radical nephroureterectomy with bladder cuff excision was performed without compromising oncological principles. Avoidance of entry into the urinary tract during surgery was taken into consideration in order to prevent tumor seeding in both open and laparoscopic nephroureterectomy cases. Approach to distal ureter was either performed with open or endoscopic techniques. Lymph node dissection was performed in case of clinical or radiological suspicion for metastasis. The demographic and clinicopathological features such as age, sex, tumor localization, distant metastasis, and concomitant tumor development in the urinary system and OS were obtained from urology records. Hematoxylin-eosin–stained slides were revised and histological grade, stage, differentiation, PNI, LVI, necrosis, LNM, and the status of the surgical margins were noted. World Health Organization (WHO) 2016 classification of urinary tumors was used for tumor grading and staging (17).

### 2.1. Statistical analysis

The correlation between OS and disease-free survival (DFS) and age, tumor size, tumor grade, tumor stage, tumor differentiation, concomitant UC, surgical margin, PNI, LVI, LNM, necrosis, and distant organ metastasis were investigated using Kaplan–Meier method, and log rank analysis. Multivariate analyses of OS were performed using the Cox regression method. P < 0.05 was considered to be the level of statistical significance. The OS and DFS of all patients during follow-up were assessed and statistical analysis was performed with SPSS version 24 (IBM Corp.; Armonk, NY, USA). 

## 3. Results

Seventy cases (94.6%) underwent open nephroureterectomy whereas four cases (5.4%) had laparoscopic nefroureterectomy. Thirty-eight (51.4%) tumors were located in the pelvis, 7 (9.5%) in the ureter, and 29 (39.2%) both in the pelvis and ureter. Sixty (81.1%) patients were male and 14 (18.9%) were female. The ages at the time of diagnosis ranged from 40 to 84 years with a median age of 63.8 years. The follow-up time of all patients was 43.5 months (±48.7) (min: 1 month-max: 204 months). Twenty-seven cases (36.5%) were ≥70 years of age whereas 47 cases (63.5%) were <70. Fifty-six (75.7%) cases were alive; however, 18 (24.3%) patients were found to be dead. The mean tumor size was 5.4 cm (0.3–17 cm). pTa, pT1, pT2, pT3, and pT4 tumors were reported in 16 (21.6%), 13 (17.6%), 4 (5.4%), 28 (37.8%), and 13 (17.6%) patients, respectively. Histopathologically, 17 cases (23%) were low-grade and 57 cases (77%) were high-grade (Table 1).

**Table 1:** Univariate analysis of demographic, clinical, and pathological characteristics for overall survival.

Clinicopathologic factors	Category	n (%)	P-values
Median age in years (range)	63.8 (min 40-max 84) (SD ± 8.9)	74	
	Age ≥ 70	27 (36.5)	0.77
	Age <70	47 (63.5)	
Tumor size	5.4 cm (min 0.3–max 17 cm)		0.21
Sex	Male	60 (81.1)	0.11
	Female	14 (18.9)	
Survival	Live	56 (75.7)	
	Ex	18 (24.3)	
Initial surgery	Open nefroureterectomy	70 (94.6)	
	Laparoscopic nefroureterectomy	4 (5.4)	
Tumor location	Renal pelvis	38 (51.4)	0.324
	Ureter	7 (9.5)	
	Renal pelvis and ureter	29 (39.2)	
Pathological T stage	pTa	16 (21.6)	0.19
	pT1	13 (17.6)	
	pT2	4 (5.4)	
	pT3	28 (37.8)	
	pT4	13 (17.6)	
Tumor grade	High	57 (77)	0.14
	Low	17 (23)	
Differentiation	Absence	52 (70.3)	<0.001
	Presence	22 (29.8)	
	Squamous	19 (25.7)	
	Sarcomatoid	2 (2.7)	
	Glandular	1 (1.4)	
Lymph node metastasis	Absence	64 (86.5)	0.042
	Presence	10 (13.5)	
Lymphovascular invasion	Absence	49 (66.2)	0.74
	Presence	25 (33.8)	
Perineural invasion	Absence	65 (87.8)	0.13
	Presence	9 (12.2)	
Tumor necrosis	Absence	45 (60.8)	<0.001
	Presence	29 (39.2)	
Surgical margins	Negative	61 (82.4)	0.28
	Positive	13 (17.6)	
Synchronous tumor	Absence	42(56.8)	0.45
	Presence	32 (43.2)	
	Bladder	31 (41.9)	
	Bladder and contralateral kidney	1 (1.4)	
Recurrent disease	Absence	68 (91.9)	0.57
	Presence	6 (8.1)	
Metastasis	Absence	58 (78.4)	<0.001
	Presence	16 (21.6)	

Twenty-two cases (29.7%) showed variant differentiation (19 squamous, 2 sarcomatoid, 1 glandular differentiation). 

Necrosis, LVI, PNI, and LNM was observed in 29 (39.2%), 25 (33.8%), 9 (12.2%), and 10 (13.5%) cases, respectively. Furthermore, 13 cases (17.6%) showed positive surgical margin (Table 1).

According to the follow-up data, 6.3% of cases with stage pTa, 23.1% of cases with stage pT1, 35.7% of cases with stage pT3, and 30.8% of the cases with stage pT4 were dead. All the cases with stage pT2 were alive. There was no statistically significant correlation between stage, tumor localization, positive surgical margin, and OS (P = 0.19, P = 0.324, P = 0.28, respectively).

Two of 17 low-grade carcinomas (11.8%) and 16 of 57 high-grade carcinomas (28.1%) were dead and no statistically significant correlation was found between tumor grade and OS (P = 0.14).

Concomitant bladder tumor was detected in 31 cases (41.9%). The tumor was located in bladder and contralateral kidney in one case (1.4%). However, the association between prognosis and concomitant urothelial cancer was not significant (P = 0.45).

**Figure 1:**
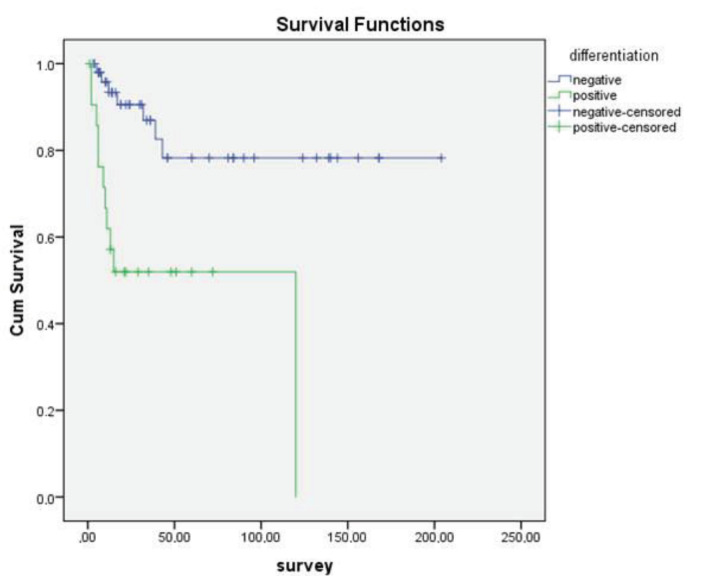
Kaplan–Meier curves of overall survival stratified according to the histological differentiation of tumors.

**Figure 2:**
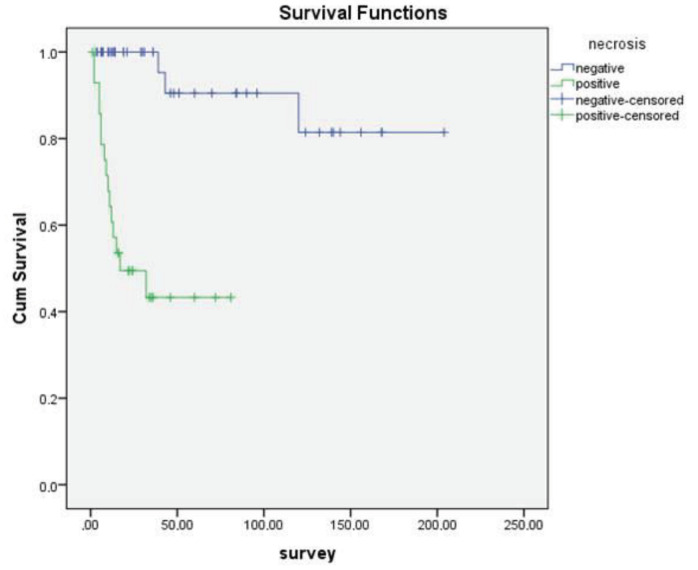
Kaplan–Meier curves of overall survival stratified according to tumor necrosis.

**Figure 3:**
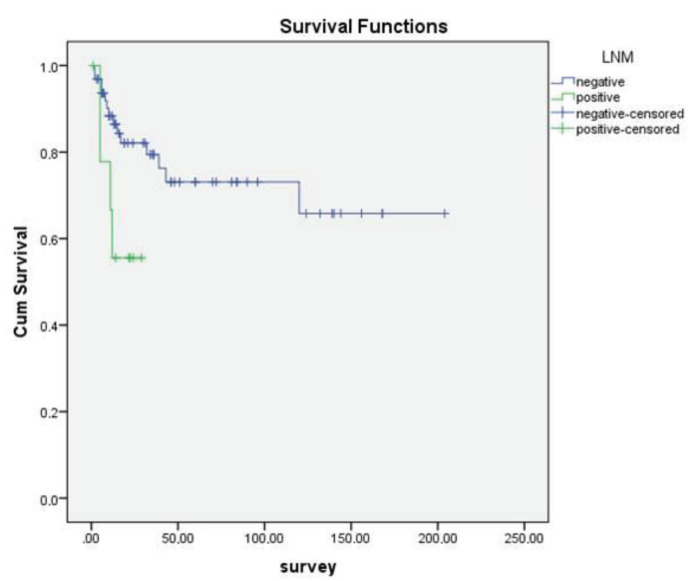
Kaplan–Meier curves of overall survival stratified according to lymph node metastasis. (LNM)

**Figure 4:**
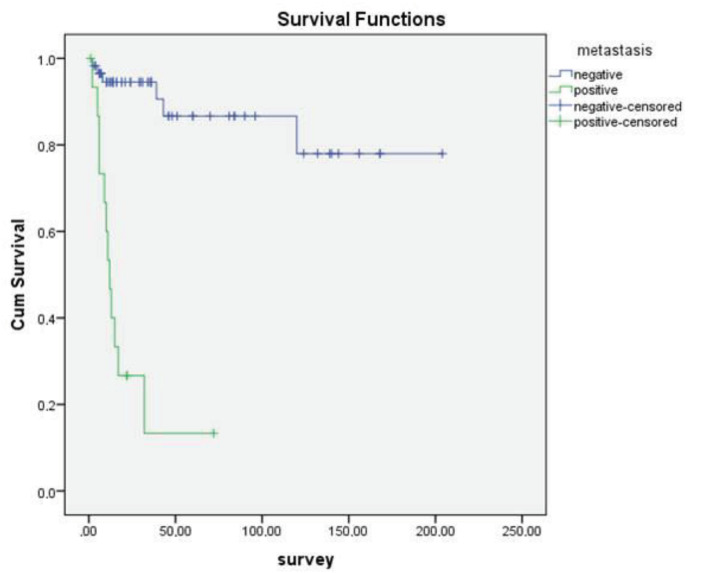
Kaplan–Meier curves of overall survival stratified according to distant metastasis.

In addition, no statistically significant correlation was found between sex, age over or under 70, tumor size, PNI, LVI, and OS (P = 0.11, P = 0.774, P = 0.21, P = 0.13, P = 0.74, respectively). However, statistically significant association was observed between OS and differentiation (Figure 1), necrosis (Figure 2), LNM (Figure 3), and distant metastasis (Figure 4) (P < 0.001, P < 0.001, P < 0.001, P = 0.042, respectively).

There was no significant correlation between synchronous tumor in the bladder (p=0.45) and OS as well as distant metastasis, differentiation, necrosis, LNM, and LVI (P = 0.96, P = 0.43, P = 0.79, P = 0.64, P = 0.92, respectively). Only distant metastasis was statistically associated with OS by multivariate analysis (P = 0.037). Table 2 demonstrates the multivariate analysis of parameters affecting OS.

**Table 2:** Multivariate analysis of parameters predicting overall survival.

	Levels	Hazard ratio	95% Cl Lower bound	95% Cl Upper bound	P-value
Tumor necrosis	NegativePositive	5.483	0.895	33.583	0.066
Tumor differentiation	NegativePositive	1.825	0.627	5.314	0.270
Lymph node metastasis	NegativePositive	1.100	0.330	3.666	0.877
Metastasis	NegativePositive	4.200	1.087	16.227	0.037

Six of 74 cases had recurrence and 2 of these 6 cases were dead. We observed no significant relationship between recurrence and OS (P = 0.57). 

The pathological stages of these recurrent cases were found as pTa (n=1), pT3 (n=4), and pT4 (n = 1). Five cases were high-grade whereas one case was low-grade. Two recurrent cases had LVI, concomitant bladder tumor and metastasis. None of them showed PNI. One case had tumor positive surgical margins. 

We found no significant relationship between DFS and age (over/under 70), stage, tumor grade, LVI, PNI, surgical margin positivity, concomitant bladder tumor and metastasis. (P = 0.711, P = 0.436, P = 0.549, P = 0.918, P = 0.393, P = 0.900, P = 0.207, P = 0.100, respectively).

Three of 45 cases without necrosis had recurrence in addition to three cases with necrosis. Three of 52 cases without any additional differentiation had recurrence. Three tumors with additional differentiation had recurrent disease. None of the cases with recurrent disease had lymph node metastasis. Four cases with no metastasis and 2 cases with metastasis had recurrent disease. No significant relationship was observed between DFS and necrosis, differentiation and lymph node involvement (P = 0.254, P = 0.103, P = 0.458, respectively). 

## 4. Discussion

UUSUC is rare but a potentially lethal disease (7). Upper urinary system tumors are generally multifocal affecting all urinary system lined by urothelium (1,9). The mean age of patients is reported as 65 years and the disease is more common in men (8). Various studies focused on the effect of clinical and pathologic parameters on UUSUC outcomes (1,5,7,10,12,18–24). Several studies reported tumor stage and grade as prognostic predictors in UUSUC (1,5,7,10,18–24).

In two different studies of Kikucki et al. and Bolenz et al., LVI was reported as an independent prognostic factor of DFS in UUSUCs (6,25). In our study, LVI was observed in 25 (33.8%) of 70 patients. There was no statistically significant correlation between LVI and OS. The presence of LVI should be stated in pathology reports in order to follow up the patients. (6).

Green et al. reported that the prognosis of bladder urothelial carcinoma is worse in women (10). Two multiinstitutional analyses performed by Fernandez et al. and Shariat et al. did not show any difference in pathologic characteristics and outcomes between sexes in UUSUC (1). Emamekhoo et al. reported that sex had no significant effect on OS and DFS of 454 cases (5). Most of our cases were men (n = 60) and no significant correlation was observed between sex and OS.

The studies about tumor localization and prognosis reported that ureteral tumors have a worse prognosis than renal pelvic tumors (1,5,7,18). The reason of this correlation is controversial while stage and treatment options may change (10). The protective effect of the renal parenchyma is thought to be associated with this finding. Also, the presence of a thin layer of adventitia surrounding the ureter, which contains an extensive plexus of blood and lymphatic vessels makes the invasion of tumor easier (21). However, several studies did not confirm the independent prognostic impact of tumor location on survival and showed the same recurrence-free survival and cancer-specific survival rates for renal pelvis and ureteral tumors (1,5). Similarly, we did not find a significant relation between OS and tumor localization. Nevertheless, this may be due to the unequal distribution of the cases for tumor localization.

Histopathologically, UUSUCs are generally high-grade tumors (9). Pathological tumor stage and histological grade are accepted as main indicators for prognosis similar to other malignant tumors (7,9,12,14,19–23). Most of the cases were high-grade in our study. Sixteen of 57 high-grade carcinomas were dead while only 2 of 17 low-grade carcinomas were found to be dead.

There was a correlation between grade or stage and OS; however, it was not statistically significant and this may be explained by the distribution numbers among the groups (P = 0.14, P = 0.19). 

The presence of tumor necrosis is an indicator of aggressiveness in almost all malignancies (1,9,12). However, recent studies reported controversial results about the prognostic role of tumor necrosis in UUSUC (1). In the study of Zhang et al., tumor necrosis was found in 48 of 100 cases and was related to pathological stage, higher tumor grade, LNM, and LVI (12). Seitz et al. detected tumor necrosis in 165 of 754 cases (21.9%) from 9 different centers and showed that the prevalence of tumor necrosis increased as the pathological stage increased. Also, they reported that tumor necrosis was related to higher grade, LNM, LVI, sessile tumoral architecture and concomitant carcinoma in situ among UUSUC. However, tumor necrosis was not an independent predictor of clinical outcome (24). In our study, necrosis showed significant relation in univariate analysis; however, this relation was not proven by multivariate analysis (P < 0.001, P = 0.66, respectively). Larger studies are needed to prove this correlation. 

Lymph node involvement is generally accepted as an important prognostic factor (1). The lymph node status remains unknown because no lymphadenectomy procedures were done in many nephroureterectomy operations (5). Our clinical approach is the excision of lymph nodes when palpable lymph nodes were observed during the operation or when preoperative radiological studies reported a suspect of lymph node positivity. 

However, there are many studies showing that LNM is an independent prognostic factor of UUSUC (1,7,12). The effect of lymph node dissection on survival is still controversial in nephroureterectomy for UUSUCs (26,27). In the metaanalysis of Guo et al., patients with LNM had worse prognosis (3). On the other hand, in the same study, it was reported that lymphadenectomy showed no significant difference on survival and recurrence in pN0 or pNx cases (3).

We observed a significant association between lymph node involvement and OS in univariate analysis; however, this correlation was not proven by multivariate analysis (P = 0.042, P = 0.877).

In the literature, distant metastasis is related to prognosis in UUSUC (12,28). We also found a significant relation between distant metastasis and OS by univariate and multivariate analysis (P < 0.001, P = 0.037).

Even though the prognostic role of squamous differentiation is accepted in UUSUC, the clinical importance is still controversial (7,13,16). Most of the studies revealed that squamous differentiation was related to higher tumor grade and advanced tumor stage in univariate analysis (13). Makise et al. observed that squamous differentiation was the most common histological variant among 140 primary UUSUC and related with poor prognosis in univariate analysis (13). Qin et al. suggested that differentiation was a poor prognostic factor especially in ureteral tumors (7). We observed that squamous differentiation is the most common variant and it showed a statistically significant correlation with OS but this was not proven by multivariate analysis (P < 0.001, P = 0.27, respectively).

The history of a bladder tumor is reported as a poor prognostic factor in UUSUC in the literature and such cases must be under more stringent follow-up regimens or being treated more aggressively (1,29,30). There are studies suggesting the effect of synchronous or metachronous bladder cancer on recurrence and survival among UUSUC (7). Novara et al. also reported that the presence of concomitant muscle invasive bladder cancer is a poor prognostic factor (23). Bladder was the most common localization of concomitant tumors (n = 8) in our study. One case had tumor in both bladder and kidney. However, the presence of concomitant bladder tumor had no effect on prognosis. 

It was reported that the presence of bladder cancer before the diagnosis of UUSUC has no significant effect on prognosis (31). We could not subgroup our cases according to the occurrence time of the previous bladder tumor.

There are some studies supporting that cisplatin-based additional treatments after surgery prolongs survival in UUSUCs (4). In another study, adjuvant chemotherapy after surgery was found to be associated with longer cancer specific and recurrence-free survival in patients with pT3N0M0 UUSUCs (32). The metastatic cases in our study group had chemotherapy.

This study has several limitations that need to be considered in interpreting the findings. The first limitation is the retrospective nature of the study. Additionally, the number of patients and the follow-up period are not enough to fully interpret the results. Finally, surgical procedures were performed by different surgeons at different institutions, explaining both the variability of intraoperative management and extent of lymph node dissection. Despite these limitations, this study showed that squamous differentiation, lymph node metastasis, distant organ metastasis, and tumor necrosis have statistically significant correlation with OS by univariate analysis in patients with UUSUC. Nevertheless, distant metastasis was the only statistically significant prognostic factor of OS observed in multivariate analysis.

Finally, well-designed and larger multiinstitutional studies are still needed to provide stronger evidence and to promote the use of these prognostic factors in the management of the treatment.
